# Inhibition of mast cell tryptase attenuates neuroinflammation via PAR-2/p38/NFκB pathway following asphyxial cardiac arrest in rats

**DOI:** 10.1186/s12974-020-01808-2

**Published:** 2020-05-04

**Authors:** Umut Ocak, Pinar Eser Ocak, Lei Huang, Weilin Xu, Yuchun Zuo, Peng Li, Marcin Gamdzyk, Gang Zuo, Jun Mo, Guangyu Zhang, John H. Zhang

**Affiliations:** 1 Department of Physiology and Pharmacology, Loma Linda University School of Medicine, Loma Linda, CA 92350 USA; 2 Department of Emergency Medicine, Bursa Yuksek Ihtisas Training and Research Hospital, University of Health Sciences, 16310 Bursa, Turkey; 3 Department of Emergency Medicine, Bursa City Hospital, 16110 Bursa, Turkey; 4 Department of Neurosurgery, Uludag University School of Medicine, 16069 Bursa, Turkey; 5 Department of Neurosurgery, Loma Linda University School of Medicine, Loma Linda, CA 92350 USA; 6 Department of Neurosurgery, The Second Affiliated Hospital, Zhejiang University School of Medicine, Zhejiang, 310009 Hangzhou China; 7 Department of Neurosurgery, The Third Xiangya Hospital, Central South University, Changsha, 410013 Hunan China; 8 Department of Neurosurgery, The Affiliated Taicang Hospital, Soochow University, Suzhou, Taicang, 215400 Jiangsu China; 9 Department of Neurosurgery, The Fourth Affiliated Hospital, Zhejiang University School of Medicine, Yiwu, Zhejiang, 322000 China; 10 Mass Spectrometry Core Facility, Loma Linda University School of Medicine, Loma Linda, CA 92350 USA; 11 Department of Anesthesiology, Loma Linda University School of Medicine, Loma Linda, CA 92350 USA; 12 Department of Neurology, Loma Linda University School of Medicine, Loma Linda, CA 92350 USA

**Keywords:** Asphyxia, Cardiac arrest, Cognitive, Global brain ischemia, Mast cell, Neuroinflammation, PAR-2, Tryptase

## Abstract

**Background:**

Cardiac arrest survivors suffer from neurological dysfunction including cognitive impairment. Cerebral mast cells, the key regulators of neuroinflammation contribute to neuroinflammation-associated cognitive dysfunction. Mast cell tryptase was demonstrated to have a proinflammatory effect on microglia via the activation of microglial protease-activated receptor-2 (PAR-2). This study investigated the potential anti-neuroinflammatory effect of mast cell tryptase inhibition and the underlying mechanism of PAR-2/p-p38/NFκB signaling following asphyxia-induced cardiac arrest in rats.

**Methods:**

Adult male Sprague-Dawley rats resuscitated from 10 min of asphyxia-induced cardiac arrest were randomized to four separate experiments including time-course, short-term outcomes, long-term outcomes and mechanism studies. The effect of mast cell tryptase inhibition on asphyxial cardiac arrest outcomes was examined after intranasal administration of selective mast cell tryptase inhibitor (APC366; 50 μg/rat or 150 μg/rat). AC55541 (selective PAR-2 activator; 30 μg/rat) and SB203580 (selective p38 inhibitor; 300 μg/rat) were used for intervention. Short-term neurocognitive functions were evaluated using the neurological deficit score, number of seizures, adhesive tape removal test, and T-maze test, while long-term cognitive functions were evaluated using the Morris water maze test. Hippocampal neuronal degeneration was evaluated by Fluoro-Jade C staining.

**Results:**

Mast cell tryptase and PAR-2 were dramatically increased in the brain following asphyxia-induced cardiac arrest. The inhibition of mast cell tryptase by APC366 improved both short- and long-term neurological outcomes in resuscitated rats. Such behavioral benefits were associated with reduced expressions of PAR-2, p-p38, NFκB, TNF-α, and IL-6 in the brain as well as less hippocampal neuronal degeneration. The anti-neuroinflammatory effect of APC366 was abolished by AC55541, which when used alone, indeed further exacerbated neuroinflammation, hippocampal neuronal degeneration, and neurologic deficits following cardiac arrest. The deleterious effects aggregated by AC55541 were minimized by p38 inhibitor.

**Conclusions:**

The inhibition of mast cell tryptase attenuated neuroinflammation, led to less hippocampal neuronal death and improved neurological deficits following cardiac arrest. This effect was at least partly mediated via inhibiting the PAR-2/p-p38/NFκB signaling pathway. Thus, mast cell tryptase might be a novel therapeutic target in the management of neurological impairment following cardiac arrest.

## Background

Sudden cardiac arrest (CA) is the leading cause of death worldwide. Although survival is increasing due to the improvements in bystander interventions such as cardiopulmonary resuscitation and defibrillation as well as post-resuscitation medical care, only a minor portion of the patients can be discharged from the hospital, albeit with moderate to severe functional impairment [1, 2]. The high mortality rate and severe functional disability following CA have been attributed to post-CA brain injury as a part of the post-CA syndrome [3].

Global cerebral ischemia-reperfusion injury (GCI) that occurs during the arrest period and subsequent return of spontaneous circulation (ROSC), trigger deleterious injury mechanisms including neuroinflammation [4] leading to irreversible brain damage [5]. Besides many other neurofunctional impairments [6], damage to the ischemia vulnerable brain regions such as hippocampus results in cognitive dysfunction following CA [7–9]. Cognitive impairment may persist up to 3 years, thereby hindering patients’ daily activities and decreasing their quality of life [10]. Although a great many studies have focused on targets that can alleviate cognitive debilitation in CA survivors, effective strategies are currently lacking.

Recently, brain mast cells (MCs), one of the key regulators of neuroinflammation [11], have been implicated in cognitive dysfunction associated with surgery, Alzheimer’s disease, and mastocytosis [12–14]. Although neuroinflammation is also underlined in GCI from which ensues hippocampal neuron degeneration [15], the contribution of MCs to neuroinflammation mediated cognitive dysfunction in the setting of CA is yet to be elucidated.

Previous studies showed that MC-tryptase, the major secretory protein of MCs, potentiates the release of proinflammatory cytokines from peripheral mononuclear cells [16]. A similar proinflammatory effect of MC-tryptase on microglia was demonstrated in vitro, in which MC-tryptase activated microglia via the protease-activated receptor-2 (PAR-2) [17]. PAR-2 is a unique member of the PAR family as it is activated by trypsin and MC-tryptase, while the rest of the PAR isoforms (PAR-1, 3, and 4) are activated by thrombin [18]. In response to MC-tryptase-mediated activation of microglial PAR-2, mitogen-activated protein kinases were phosphorylated leading to the release of microglia-derived proinflammatory cytokines [17].

**Fig. 1:**
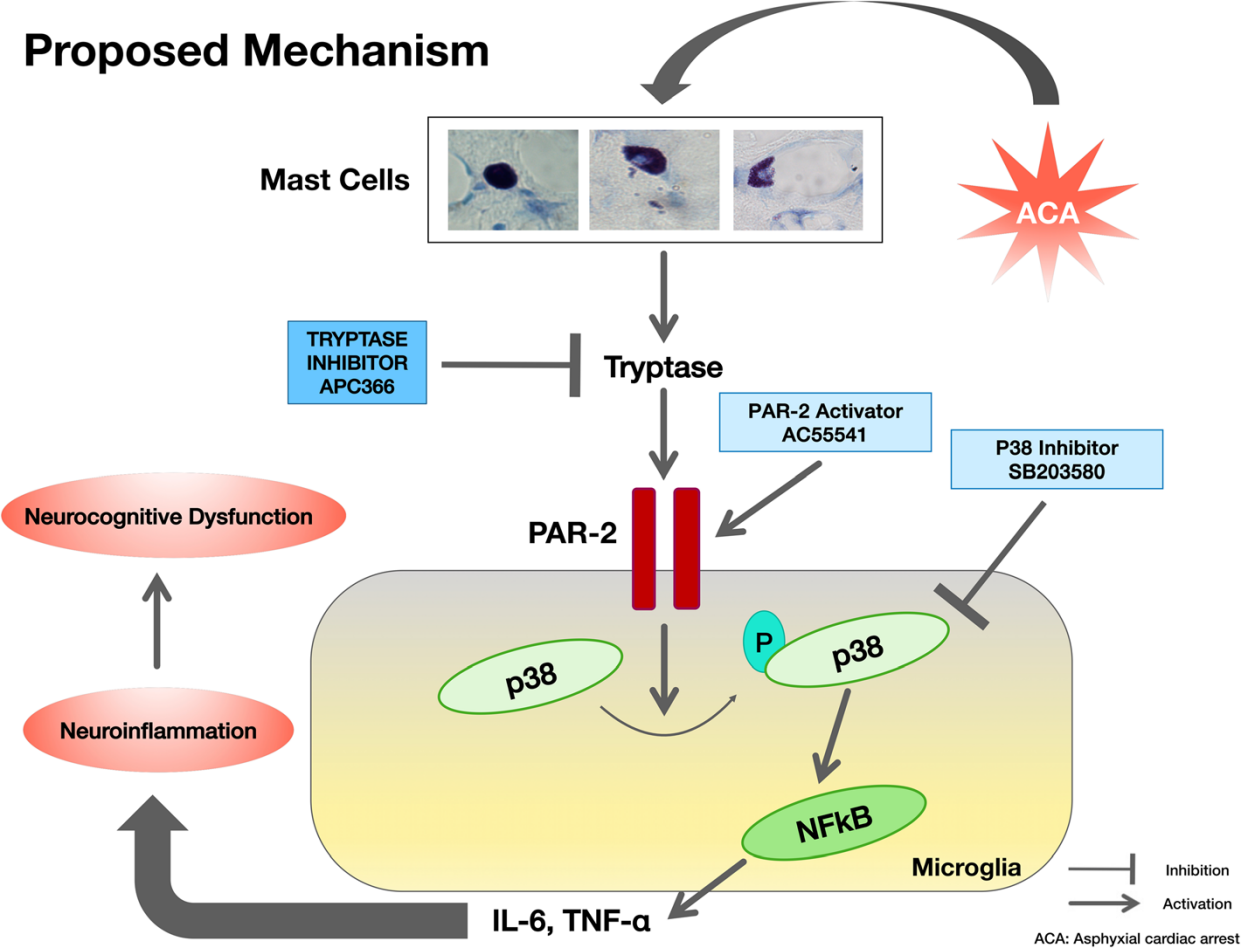
The schematic for the proposed mechanism underlying the anti-neuroinflammatory effect of MC-tryptase inhibition. We propose that following ACA, MCs will be activated and degranulated leading to the release of MC-derived tryptase in the brain, which will activate microglial PAR-2. Phosphorylation and activation of p38 and activation of NFκB in response to activated PAR-2 will result in the release of microglia-derived proinflammatory cytokines, IL-6, and TNF-α. Resultantly, neuroinflammation will contribute to neurocognitive dysfunction following ACA. For this study, we used selective MC-tryptase inhibitor APC366 for treatment purposes while PAR-2 activator AC55541 and p-38 inhibitor SB203580 were used for intervention

Based on the abovementioned results, we hypothesized that the inhibition of MC-tryptase would reduce neuroinflammation, leading to less hippocampal neuronal degeneration, and improve neurologic functions including cognitive functions following asphyxia-induced CA (ACA) in rats. This anti-neuroinflammatory effect may occur partly via inhibiting the PAR-2/phosphorylated p38 (p-p38)/NFκB signaling pathway. The proposed mechanism for the current study is presented in Fig. 1.

## Methods

### Animals and rat model of ACA

All experimental procedures were approved by the Institutional Animal Care and Use Committee at Loma Linda University, Loma Linda, CA, USA. All experiments were in concordance with the National Institutes of Health Guide for the Care and Use of Laboratory Animals and reported according to the ARRIVE guidelines.

Adult male Sprague-Dawley rats (450–500 g, Envigo, IN, USA) were housed in a humidity and temperature-controlled facility with 12 h light/dark cycle and ad libitum food and water access. The rat model of ACA was performed as previously described [19–21]. Briefly, under deep anesthesia with pentobarbital (intraperitoneal, 45 mg/kg, Virbac AH, Inc., Fort Worth, TX, USA), rats were endotracheally intubated with a 14-gauge plastic catheter under laryngoscopy. The left femoral artery and vein were exposed via 1 cm skin incision along the left groin followed by blunt dissection of the surrounding connective tissue. A PE 50 catheter (Becton Dickinson, Franklin Lakes, NJ, USA) was inserted in the femoral artery and connected to a pressure transducer. Another PE 50 catheter was inserted in the femoral vein for drug administration. A rectal probe (Model BAT-12, Physitemp Instrument Inc., Clifton, NJ, USA) was inserted to monitor rectal body temperature. Lead-II electrocardiogram was recorded. Rats were mechanically ventilated (respiratory frequency 100 bpm, tidal volume 0.55 mL/100 g, FiO2 21%) for 15 min before ACA induction. ACA was induced by chemical neuromuscular blockade with intravenous vecuronium (2 mg/kg; Mylan Institutional LLC., Rockford, IL, USA) followed by disconnecting the ventilator and obstructing the intubation tube. After 10 min of asphyxia, resuscitation was initiated by unclamping the tracheal tube, administering epinephrine (7.5 μg/kg; International Medication System, LLC., South El Monte, CA, USA) and sodium bicarbonate (1 mEq/kg; Hospira, Lake Forest, IL, USA), applying precordial compressions with a pneumatically driven mechanical chest compressor and coincident mechanical ventilation with 100% oxygen at a ratio of 2:1. Successful resuscitation and ROSC were defined as mean arterial pressure of > 60 mmHg and return of sinus rhythm for 5 min. Rats were excluded from the study if ROSC was achieved beyond 5 min of resuscitation or if ROSC was not achieved. After ROSC, mechanical ventilation was continued with 100% oxygen for 30 min and gradually reduced to 21% every 10 min within 1 h. At 1 h post-resuscitation, the animals were weaned from the ventilator, extubated, and all catheters were removed. The wound was sutured, and the animals were allowed to recover. A heating lamp was used to maintain the body temperature at 36.5 ± 0.5 °C. Shams underwent the same surgical procedures and baseline ventilation without ACA induction. Electrocardiogram, end-tidal carbon dioxide, and arterial blood pressure were continuously recorded from 15 min before asphyxia induction until 1 h after ROSC on a PC-based data-acquisition system supported by the WINDAQ software (DATAQ, Akron, OH, USA). The baseline and post-resuscitation hemodynamics of rats are presented in Table 1.

**Table 1:** Baseline and post-resuscitation hemodynamics and the resuscitation time of the animals

Parameters	Baseline	Post-ROSC 5 min	Post-ROSC 30 min	Post-ROSC 60 min
Body weight (g)	487.7 (± 10.4)	–	–	–
MAP (mmHg)	123.7 (± 14.9)	121.2 (± 22)	103.1 (± 11.5)*	114.4 (± 11.7)
Hearth rate (bpm)	277 (± 35.5)	300 (± 22.1)*	288.9 (± 24.3)	306.7 (± 29.3)*
EtCO2 (mmHg)	45.5 (± 5.5)	56.7 (± 5.3)	53.9 (± 3.5)	50.4 (± 4.2)
CPR time (s)	109.2 (± 57.7)	–	–	–

*CPR* cardiopulmonary resuscitation, *EtCO2* end-tidal carbon dioxide, *MAP* mean arterial pressure, *ROSC* return of spontaneous circulation

Data are expressed as mean + standard deviation, *n* = 120. ANOVA, Tukey. *p < 0.05 compared to baseline

### Experimental design

**Fig. 2:**
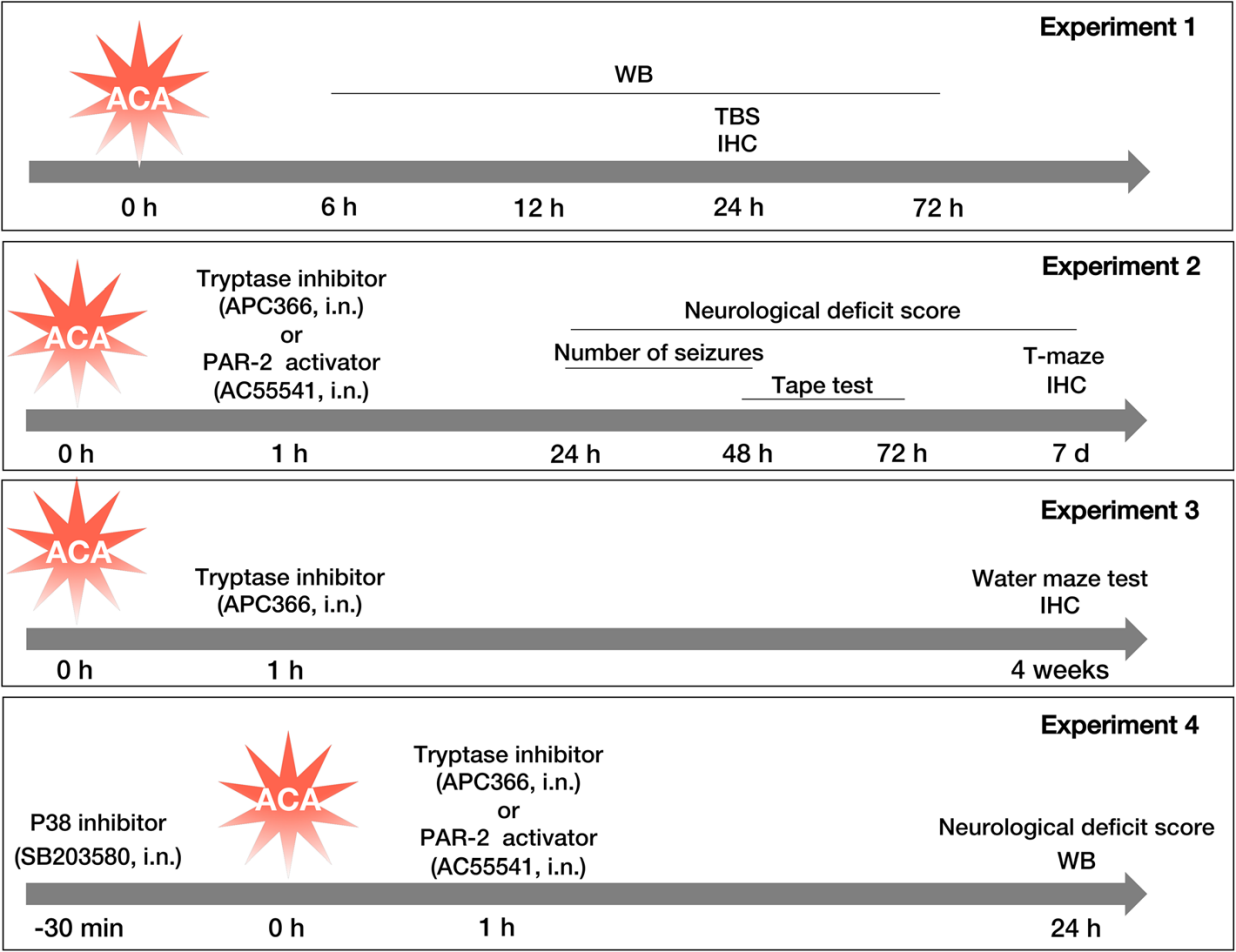
Experimental design for the present study. Four main experiments including time course (experiment 1), short-term outcomes (experiment 2), long-term outcomes (experiment 3), and mechanism studies (experiment 4) were performed. d days, h hours, IHC immunohistochemistry, i.n. intranasal, min minutes, TBS Toluidine blue staining, WB western blot

The animals were randomly assigned to four main experiments. The design of the experiments and the number and distribution of animals per experimental groups are summarized in Fig. 2 and Table 2, respectively.

**Table 2:** The number and distribution of the animals included for the present study

		Groups (*n*)	Mortality (*n*)
Time course study	WB (6, 12, 24, and 72 h post-ROSC)	Sham (*n* = 4)	0
		ACA (6 h, 12 h, 24 h, 72 h) (*n* = 16)	3 (1 died at 12 h, 1 died at 15 h, and 1 died at 22 h post-ROSC)
	Cellular localization (24 h post-ROSC)	Sham (*n* = 1), ACA (*n* = 1)	0
	Toluidine blue staining (24 h post-ROSC)	Sham (*n* = 1)	0
		ACA (*n* = 1)	0
Short-term outcome study (up to 7-day post ROSC)	Fluoro-Jade C staining	Sham (*n* = 6)	0
		ACA + vehicle (*n* = 6)	2 (1 at 24 h post-ROSC, 1 died at 48 h post-ROSC)
		ACA + APC366 (50 μg) (*n* = 6)	2 (1 died at 48 h post-ROSC, 1 died at 70 h post-ROSC)
		ACA + APC366 (150) (*n* = 6)	1 (died at 6 h post-ROSC)
		ACA + AC55541 (30 μg) (*n* = 6)	2 (1 died on 5th day post-ROSC, 1 died on 6th post-ROSC)
Long-term outcome study (30-day post-ROSC)	Fluoro-Jade C staining	Sham (*n* = 6)	0
		ACA + vehicle (*n* = 6)	0
		ACA + APC366 (50 μg) (*n* = 6)	0
Mechanism study (24 h post-ROSC)	Western blot	Sham (*n* = 6)	0
		ACA + vehicle (*n* = 6)	0
		ACA + APC366 (50 μg) (*n* = 6)	0
		ACA + AC55541 (30 μg) (*n* = 6)	0
		ACA + APC366 (50 μg) + AC55541 (30 μg) (*n* = 6)	1 (died at 8 h post-ROSC)
		ACA + AC55541 (30 μg) + SB203580 (300 μg) (*n* = 6)	0
	Mass spectrometry	APC366 (*n* = 1)	0
Total	**120**	**109**	**11**

*ACA* asphyxial cardiac arrest, *h* hours, *ROSC* return of spontaneous circulation

#### Experiment 1 (time course study, cellular co-localization, and Toluidine blue staining)

Endogenous expression of the pathway proteins was evaluated with western blot using whole brain samples obtained from sham (24 h) and ACA animals at different time points (6, 12, 24, and 72 h) following the injury. Cellular co-localization of PAR-2 with microglia was evaluated by double immunofluorescence staining, while MCs were evaluated by Toluidine blue staining at 24 h after ACA.

#### Experiment 2 (short-term outcome study)

The effect of MC-tryptase inhibition and PAR-2 activation on ACA outcomes was examined after intranasal administration of selective MC-tryptase inhibitor (APC366; 50 μg/rat or 150 μg/rat; Santa Cruz Biotechnology, Dallas, TX, USA) or intranasal administration of selective PAR-2 activator (AC55541; 30 μg/rat; Santa Cruz Biotechnology, Dallas, TX, USA), respectively. Neurologic functions were assessed using neurological deficit score (NDS), number of seizures, adhesive tape removal test, and T-maze test at various time points up to 7 days following ACA. The animals including the sham group were sacrificed on day 7 following ACA and the hippocampal neuron degeneration was evaluated with Fluoro-Jade C (FJC) staining. Determination of APC366 in the brain tissue using mass spectrometry was performed at 24 h after intranasal administration of the drug at dose of 50 μg/rat. Based on the results of experiment 2, APC366 at 50 μg/rat was chosen for experiments 3 and 4.

#### Experiment 3 (long-term outcome study)

The effect of MC-tryptase inhibition on long-term neurologic functions and hippocampal neuronal degeneration was evaluated at 28 days following ACA. Neurologic function was evaluated with Morris water maze test. Animals including the sham group were harvested on day 28 following ACA and hippocampal neuron degeneration was evaluated with FJC staining.

#### Experiment 4 (mechanism study)

The potential mechanism underlying neuroprotective effects of MC-tryptase inhibition through PAR-2/p-p38/NFκB pathway was evaluated with western blot at 24 h following ACA. Neurologic outcome was evaluated with NDS at 24 h following ACA. AC55541 (selective PAR-2 activator; 30 μg/rat) and SB203580 (selective p38 inhibitor; 300 μg/rat; Santa Cruz Biotechnology, Dallas, TX, USA) were used for intervention.

### Intranasal drug administration

All drugs were diluted in 20% ethanol and delivered via the intranasal route [22]. Briefly, rats were placed in supine position under 2% isoflurane anesthesia. Then, 20% ethanol (vehicle) or 2 different doses of APC366 (50 μg/rat and 150 μg/rat) or AC55541 (30 μg/rat) were administered at 1 h after ACA [23]. SB203580 (300 μg/rat) was administered 30 min before the induction of ACA [24]. A total volume of 30 μL was delivered for a period of 10 min with 5 μL into one naris and alternately into left and right nares every 2 min.

### Liquid chromatography–mass spectrometry

The liquid chromatography–mass spectrometry (LC-MS/MS) detection system which is comprised an HPLC-pump, autosampler, and Agilent 6410 Triple Quadrupole LC/MS (Agilent Technologies, CA, USA) with an atmospheric pressure chemical ionization source was used to determine the presence of APC366 (50 μg/rat) in the brain after its intranasal administration [25]. During sample preparation, 200 mg of brain tissue was homogenized and 2 mL of acetonitrile (Sigma-Aldrich, USA) was added afterwards. Then, the mixture was centrifuged at 14,000*g* for 30 min at 4 °C. The supernatant was dried under negative pressure (below 2.0 kPa) for 7 h at 4 °C and the residue was reconstituted with 1000 μL 50% acetonitrile before centrifugation at 14,000*g* for 20 min at 4 °C. A total of 20 μL of the supernatant was then injected into the LC-MS/MS system. The MS spectra were collected under the positive reflector mode from m/z 100–1000. The MS/MS spectra were acquired using a collision energy of 34 kV with the metastable suppressor on and the LC-MS/MS data was visualized and analyzed by using MassHunter software version B.08.00 (Agilent Technologies, CA, USA).

### Assessment of short-term neurologic functions

Consciousness, respiration, corneal reflex, auditory reflex, motor function, and behavior were evaluated with *NDS* at 24, 48 and 72 h and at 7 days after ACA. The total score of the test ranged between 0 (normal) and 500 (coma) with higher scores indicating worse performance in neurological testing [26]. *Number of seizures* was recorded with an observation period of 4 h/day at 24 and 48 h following ACA. *Adhesive removal test* was used to assess the sensorimotor integration at 48 and 72 h but not at 24 h as the animals were too vulnerable and seizure activity were readily trigged by any stimulation during the first day following ACA. The ability of the animals to remove the tapes which were placed on either right or left forepaws was observed for 120 s and the results were recorded as “successful” or “failed.” Following 6 consequent trials, the results were expressed as percentage of tape removal [27]. *T-maze spontaneous alternation test* was performed to assess the cognitive deficits at 7 days after ACA. The percentage of spontaneous alternation (number of turns in each goal arm) was recorded and the results were expressed as percent with respect to 50% reference [28].

### Assessment of long-term neurologic function

The Morris water maze test was performed on days 23–28 to assess long-term spatial learning capacity and reference memory [29, 30]. A circular pool (diameter 110 cm) filled with water (24 ± 1 °C) in which a transparent escape platform (diameter 11 cm) was submerged 1 cm beneath the water and placed at the epicenter of one of the quadrants was used. The cued water maze test which was performed on day 23 was used as a control to determine any potential sensorimotor and/or motivational deficits that could affect the performance of the rats during the spatial water maze test. During the spatial water maze test that was performed on days 24–27 after ACA, the rats were placed in various start locations using a semi-random method and allowed to find the escape platform in 60 s. On day 28, the platform was removed, and the rats were tested with a 60-s probe trial to assess the spatial memory retention. Swimming patterns including distance, latency, and speed as well as probe quadrant duration were traced, recorded, and analyzed via a video recording system (Noldus Ethovision; Noldus, Tacoma, WA, USA).

### Histological analyses

Under deep anesthesia, rats were perfused transcardially with ice cold phosphate buffered saline (PBS, 0.01 M, pH 7.4) followed by 10% formalin. The brains were extracted rapidly, fixed in 10% formalin at 4 °C for 24 h, and then dehydrated in 30% sucrose for another 72 h. After embedding in OCT (Scigen Scientific Gardena, CA, USA), the samples were frozen at − 80 °C and coronal brain sections (10 μm thickness) were obtained 3.8 mm posterior to the bregma using a cryostat (CM3050S; Leica Microsystems, Bannockburn, III, Germany) [31, 32].

Cellular co-localization of PAR-2 with microglia marker calcium-binding adaptor molecule-1 (Iba-1) was evaluated by double immunofluorescence staining. To this end, the slices were washed in PBS (3 × 5 min), blocked with 5% donkey serum at room temperature for 1 h, and then incubated overnight at 4 °C with the following primary antibodies (Abcam, Cambridge, MA, USA): anti-PAR-2 (1:200), anti-Iba-1 (1:200). Then, the sections were washed in PBS (3 × 5 min) and incubated with appropriate secondary antibodies (1:100, Jackson Immuno Research, West Grove, PA, USA) at room temperature for 1 h. The slides were visualized and photographed under a fluorescence microscope (BZ-X800; Keyence Coorporation, Itasca, IL, USA).

Fluoro-Jade C (FJC) staining was performed using the Fluoro-Jade C Ready-to-Dilute Staining Kit (Biosensis, USA) according to the manufacturer’s instructions to identify the degenerating hippocampal neurons. FJC-positive neurons within the hippocampus were counted with the ImageJ software (ImageJ 1.5, NIH, USA). The data were presented as the number of FJC-positive neurons in the fields as cells/mm^2^.

For Toluidine blue staining which was used for the identification of brain MCs [33], the slices were stained with fresh prepared Toluidine blue solution (0.1%, pH = 2.0) for 3 min and then washed with distillated water 3 times. The slices were dehydrated through 75%, 95%, and 2 changes of 100% alcohol, cleared in xylene substitute and coverslip with mounting medium. The slides were visualized and photographed under microscope.

### Western blot analysis

Western blotting was performed for brain expressions of MC-tryptase, PAR-2, p38, phosphorylated p38 (p-p38), NFκB, tumor necrosis factor-α (TNF-α), and interleukin 6 (IL-6) as previously described [34–36]. Briefly, rats were perfused transcardially with ice cold PBS (0.01 M, pH 7.4) under deep anesthesia followed by brain extraction. Then, whole brain samples were snap frozen in liquid nitrogen and stored in − 80 °C until use. During sample preparation, brains were homogenized in RIPA lysis buffer (Santa Cruz Biotechnology Inc., TX, USA) and centrifuged at 14,000*g* at 4 °C for 30 min. The supernatants were transferred to another tube and protein concentrations were measured for each sample using detergent compatible assay (DC protein assay, Bio-Rad Laboratories, CA, USA). Then, equal amounts of protein (30 μg) were separated by sodium dodecyl sulfate polyacrylamide gel electrophoresis and transferred onto nitrocellulose membranes (0.2 μm). The membranes were blocked with 5% non-fat blocking grade milk (Bio-Rad, Hercules, CA, USA) and incubated overnight at 4 °C with the following primary antibodies (Santa Cruz Biotechnology, Dallas, TX, USA): anti-tryptase (1:500), anti-PAR-2 (1:500), anti-p38 (1:500), anti-p-p38 (1:500), anti-IL-6 (1:500), anti-NFκB (1:500), anti-TNF-α (1:500), and anti-β-actin (1:2000). On the following day, appropriate secondary antibodies (1:2000, Santa Cruz, Dallas, TX, USA) were applied at room temperature for 2 h. The bands were visualized by ECL Plus chemiluminescence reagent kit (Amersham Bioscience, PA, USA) and relative densities of the immunoblots were analyzed using the ImageJ software (Image J 1.4, NIH, USA). Beta-actin was used as an internal control to normalize the results. The standards were prepared in the same buffer that was used for protein extraction. On each membrane, β-actin was used as loading control, whose molecular weight is 42 kDa. Because its molecular weight is similar to that of p38 and p-p38, we used the stripping method to analyze the same membrane sequentially with two antibodies by stripping p38 or p-p38 antibody from the blot and subsequently incubating it with β-actin antibody. Similar approach was used for NFkB whose molecular weight is similar to MC-tryptase. Therefore, p38 and p-p38 or tryptase or NFkB were blot on different membranes, but each membrane had β-actin as loading control for quantification purposes. The quantification value of each blot was calculated as the ratio between the density of protein of interest and the density of β-actin.

### Statistical analysis

Data were presented as mean ± standard deviation and analyzed using GraphPad Prism 7 (GraphPad Software, San Diego, CA, USA). One-way ANOVA followed by Tukey’s post hoc test was used for multiple comparisons between groups. Two-way ANOVA was used to analyze long-term neurobehavioral results. A *p* < 0.05 was considered statistically significant.

## Results

A total of 170 rats were used for the study, 50 ACA rats of which were excluded due to failed resuscitation (*n* = 42, non-ROSC) or prolonged resuscitation time (*n* = 8, CPR more than 5 min to achieve ROSC). Among the 120 rats included in the study, there were 24 shams which had 0% mortality and 96 of ACA rats which had 11.4% post-ROSC mortality (11/96 died at different time points post-ROSC) (Table 2).

### Temporal expression of endogenous MC-tryptase, PAR-2, p38, p-p38, and NFκB in the brain after ACA

**Fig. 3:**
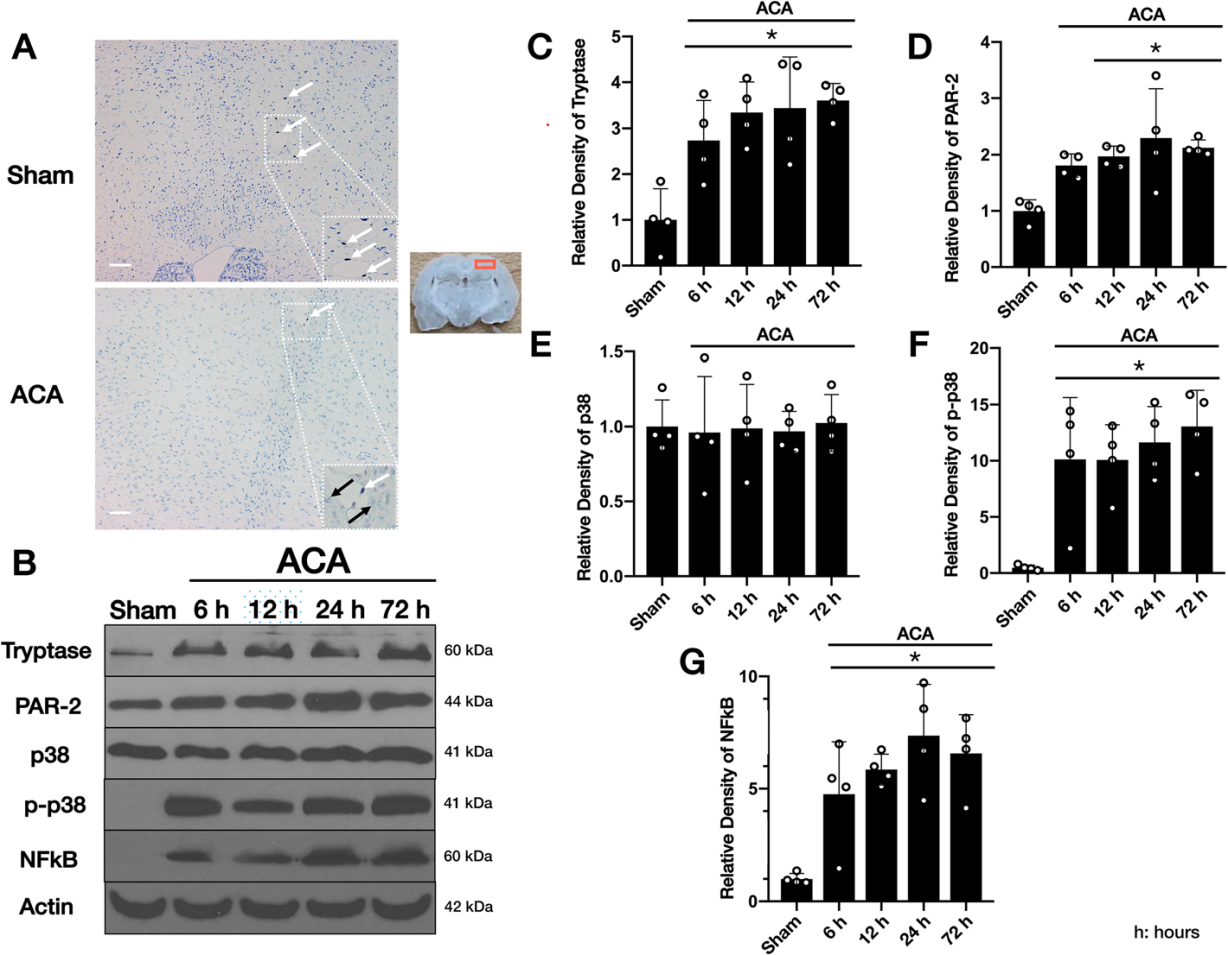
Activation of MCs and temporal expressions of MC-tryptase, PAR-2, p38, p-p38, and NFκB following ACA. Representative Toluidine blue staining microphotographs demonstrated that ACA promoted the activation and degranulation of brain MCs, leading to decreased number of intact, granulated MCs, less intensive staining, and the appearance of ghost cells at 24 h following the injury (**a**). White arrows: perivascular intact, granulated MCs. Black arrows: ghost cells. Top panel indicates the location of staining (small red box). Scale bar = 25 μm, *n* = 1/group. Representative western blot images (**b**) and quantitative analyses of endogenous MC-tryptase (**c**), PAR-2 (**d**), p38 (**e**), p-p38 (**f**), and NFκB (**g**) over 72 h following ACA. Significant increases in MC-tryptase, PAR-2, p38, p-p38, and NFκB, starting early after the injury and persisting until 72 h were observed in animals subjected to ACA compared to the sham group. P38 levels did not change over time. Data are expressed as mean ± SD. *n* = 4/group. ANOVA, Tukey. *p < 0.05 compared to sham

Toluidine blue staining showed intensive staining of perivascularly located, intact, and granulated brain MCs in the sham group. Following ACA, decreased number of intact MCs with less intensive Toluidine blue staining and the appearance of “ghost cells” suggested that ACA promoted the activation and degranulation of MCs (Fig. 3a).

Western blot consistently revealed significant increases in endogenous protein levels of MC-tryptase, PAR-2, p38, p-p38, and NFκB that started early after ACA and persisted until 72 h compared to the sham group. P38 levels did not change over time (Fig. 3b–g, Additional file 2).

**Fig. 4:**
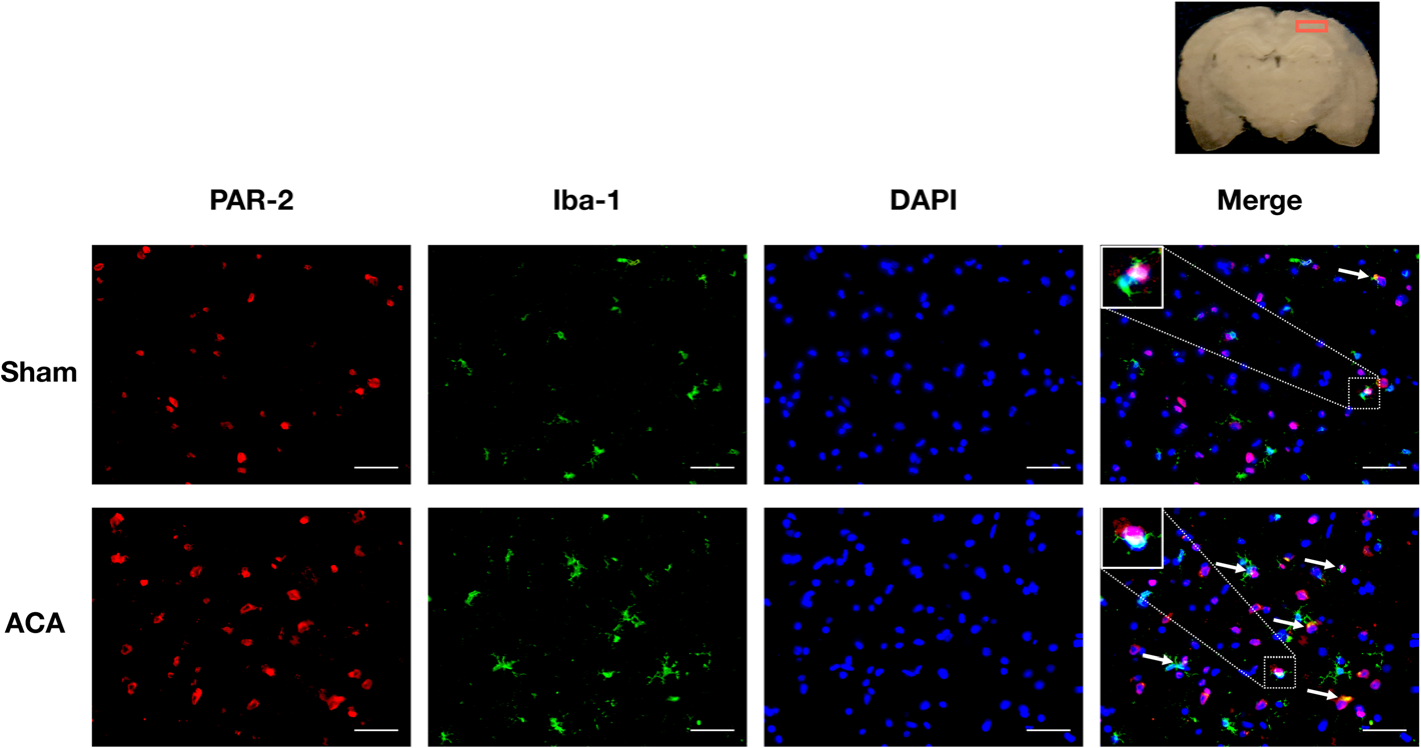
Cellular co-localization of PAR-2 in microglia. Representative double immunofluorescence staining images at 24 h following ACA showed that PAR-2 (red) co-localized with Iba-1 positive microglia (green) in both sham and ACA groups (white arrows). Top panel indicates the location of staining (small red box). Scale bar = 50 μm, *n* = 1/group

Double immunofluorescence staining revealed PAR-2 co-localizing with microglia in both sham and ACA animals at 24 h after ACA. There were more PAR-2 positive microglia in the rats subjected to ACA compared with the shams (Fig. 4).

### Inhibition of MC-tryptase ameliorated short-term neurobehavioral deficits following ACA

**Fig. 5:**
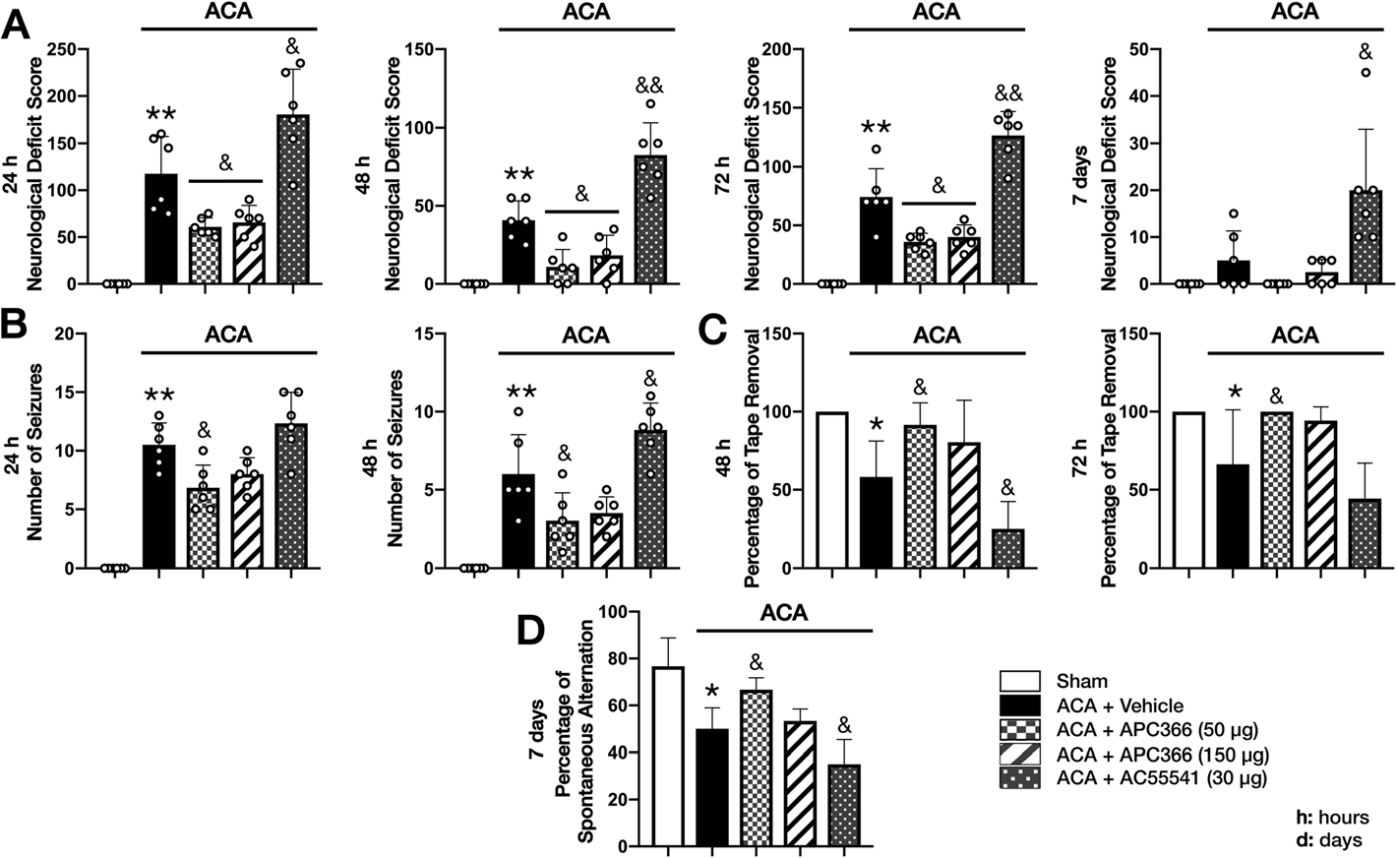
APC366 improved short-term neurological deficits after ACA. **a** Effect of APC366 on neurological deficit score (NDS) at 24, 48, and 72 h and at 7 days after ACA. **b** Seizure activity at 24 and 48 h after ACA. **c** Adhesive tape removal at 48 and 72 h after ACA. **d** T-maze test at 7 days after ACA. ACA was associated with significantly worse neurologic functions compared to the shams in all tests except 7-day NDS. APC366 treatment at both doses of 50 μg and 150 μg improved NDS at 24, 48, and 72 h after ACA compared to the vehicle-treated ACA rats. APC366 at dose of 50 μg significantly reduced the seizure activity and improved performance of adhesive tape removal and T-maze tests compared to the ACA + vehicle group. PAR-2 activation with AC55541 exacerbated ACA-induced neurobehavioral deficits in all neurobehavioral tests except for the seizure activity at 24 h and adhesive tape removal at 72 h after ACA. Data are expressed as mean ± SD. *n* = 6/group. ANOVA, Tukey. **p < 0.001 compared to sham, *p < 0.05 compared to sham, &&*p* < 0.001 compared to ACA + vehicle, &*p* < 0.05 compared to ACA + vehicle

Rats subjected to ACA exhibited significantly poorer neurologic functions compared to the sham-operated animals in all tests except NDS at 7 days. APC366 treatment at both doses of 50 μg and 150 μg significantly improved the NDS at 24, 48, and 72 h after ACA compared to vehicle-treated ACA rats. The NDS did not differ notably between 50 and 150 μg of APC366 treatment groups (Fig. 5a). However, only APC366 at a dose of 50 μg significantly reduced the seizure activity and improved the performance of the adhesive tape removal and T-maze tests compared to the ACA + vehicle group (Fig. 5b–d). AC55541 significantly exacerbated ACA-induced neurobehavioral deficits in all neurobehavioral tests except for seizure activity at 24 h and adhesive tape removal at 72 h after ACA (Fig. 5a–d).

### Inhibition of MC-tryptase reduced hippocampal neuronal degeneration at 7 days following ACA

**Fig. 6:**
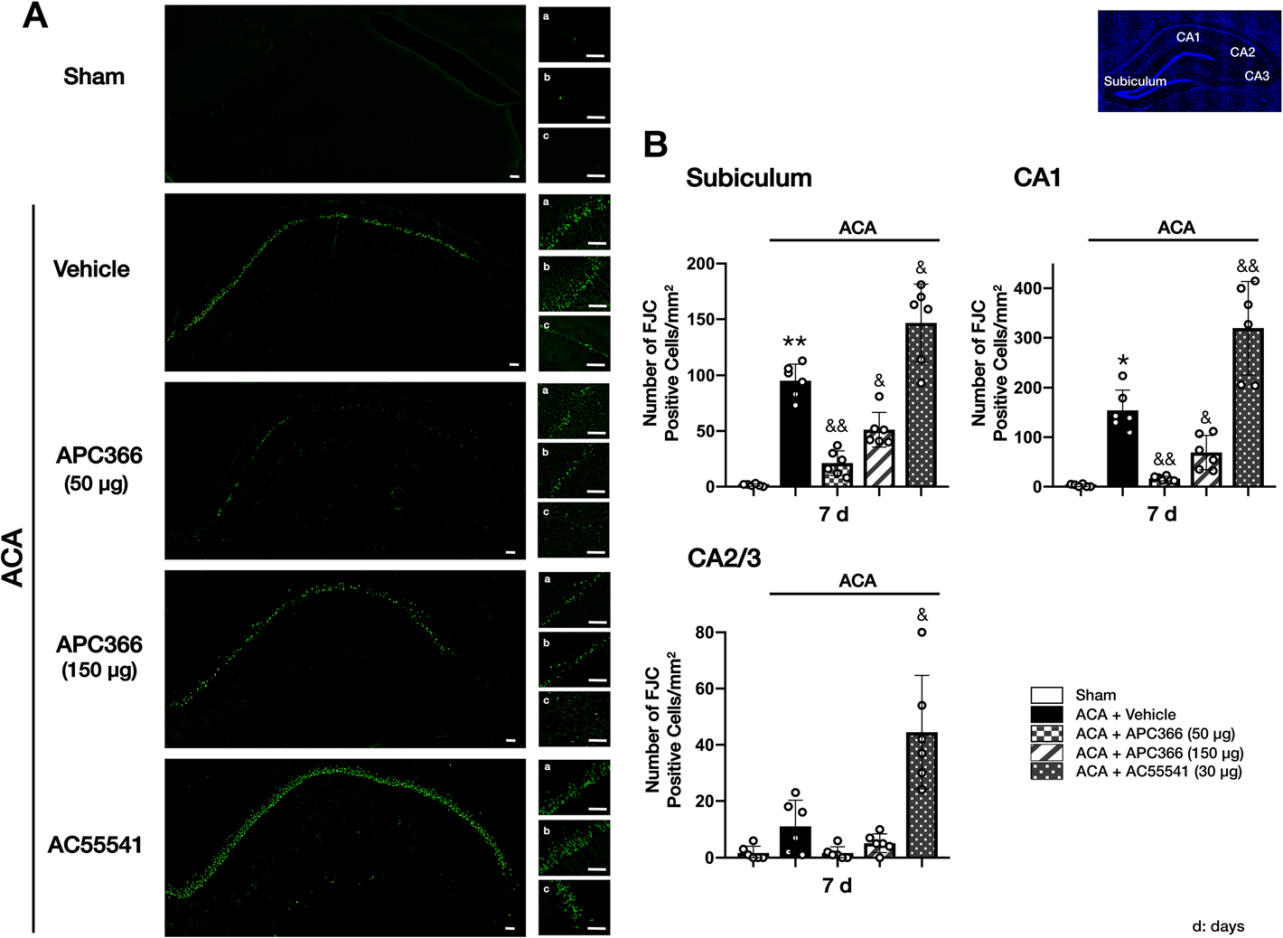
APC366 reduced FJC-positive degenerating neurons at 7 days after ACA. **a** Representative FJC staining microphotographs. **b** Quantitative analyses of FJC-positive cells in (a) hippocampal subiculum, (b) CA1, and (c) CA2/3. Scale bar = 50 μm. ACA caused significant neuronal degeneration in the subiculum and CA1 regions. The activation of PAR-2 with AC55541 further exacerbated neuronal degeneration in rats subjected to ACA. APC366 at doses of 50 μg and 150 μg significantly reduced the number of FJC-positive cells in the subiculum and CA1 regions compared to the vehicle or AC55541-treated ACA rats. The tendency toward better treatment efficacy of 50 μg APC366 than 150 μg APC366 did not reach statistical significance. Data are expressed as mean ± SD. *n* = 6/group. ANOVA, Tukey. **p < 0.001 compared to sham, *p < 0.05 compared to sham, &&*p* < 0.001 compared to ACA + vehicle, &*p* < 0.05 compared to ACA + vehicle

Fluoro-Jade C staining was performed to evaluate neuronal degeneration within the hippocampal subiculum, CA1, and CA2/3 regions at 7 days following ACA. In the subiculum and CA1 regions but not in the CA2/3, ACA significantly increased neuronal degeneration compared to the sham animals. AC55541 significantly exacerbated neuronal degeneration in all areas compared to the ACA + vehicle group. APC366 at both doses of 50 μg and 150 μg significantly reduced the number of FJC-positive cells in the subiculum and CA1 regions compared to vehicle or AC55541-treated ACA rats. There was a tendency toward better treatment efficacy of 50 μg APC366 than 150 μg APC366, but no statistical significance was reached (Fig. 6a, b).

### Inhibition of MC-tryptase improved long-term spatial learning and memory and reduced hippocampal neuronal degeneration at 28 days following ACA

**Fig. 7:**
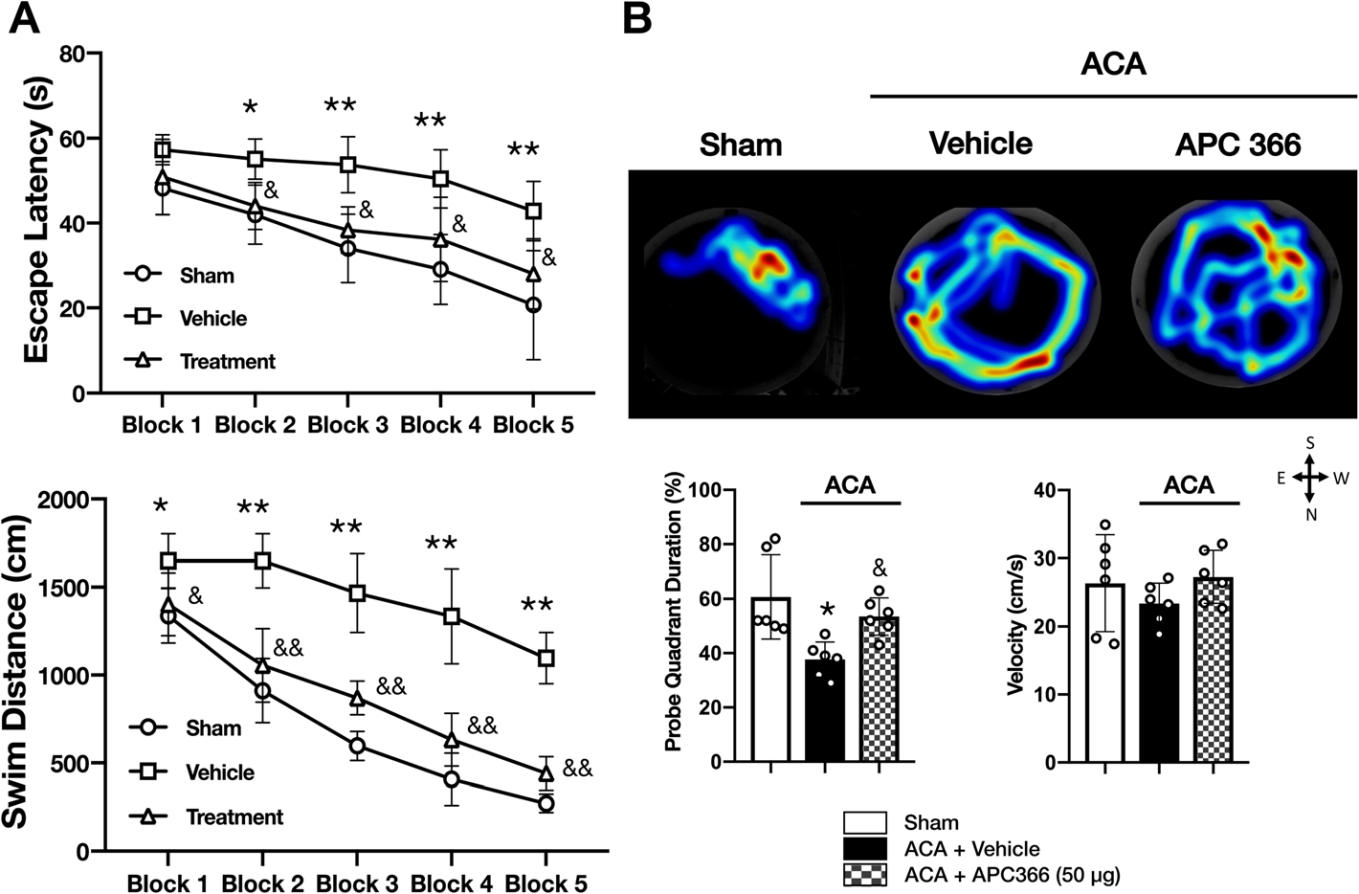
APC366 improved long-term spatial learning memory after ACA. **a** Effect of APC366 on escape latency and swimming distance of the Morris water maze test. **b** Representative heatmaps, quantification of probe quadrant duration, and swimming velocities in the probe trial of the Morris water maze test. ACA was associated with prolonged escape latency and swim distance in vehicle-treated animals compared to the sham group, which was markedly improved following APC366 treatment. APC366 treatment also significantly increased probe quadrant duration that was remarkably decreased in the ACA + vehicle group compared to the sham group in the probe trial. There was no significant difference among groups in swim velocity. Data are expressed as mean ± SD. *n* = 6/group. ANOVA, Tukey. **p < 0.001 compared to sham, *p < 0.05 compared to sham, &&*p* < 0.001 compared to ACA + vehicle, &*p* < 0.05 compared to ACA + vehicle

**Fig. 8:**
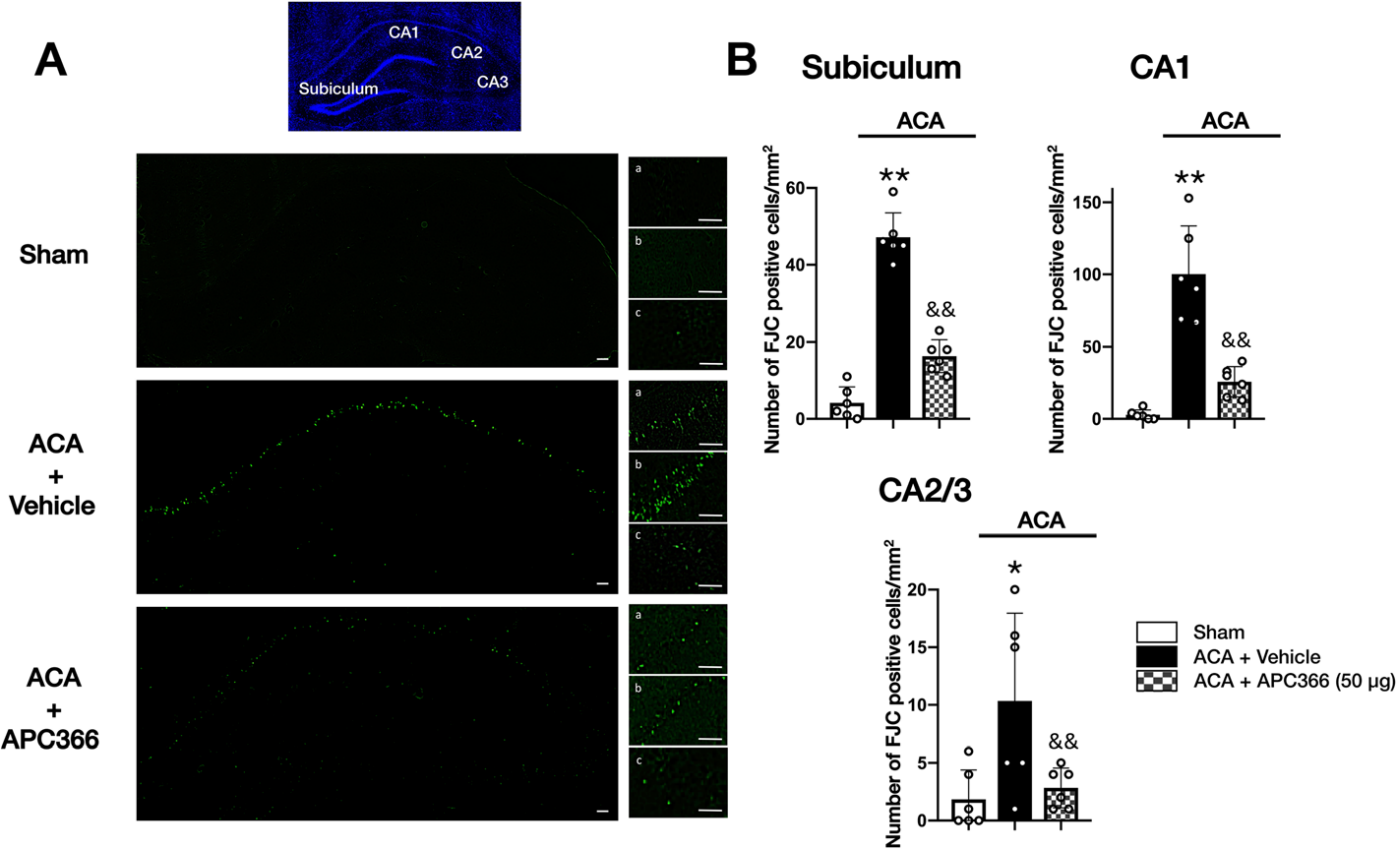
APC366 improved reduced hippocampal neuronal degeneration at 28 days after ACA. **a** Representative Fluoro-Jade C (FJC) staining microphotographs. **b** The number of FJC-positive cells in the (a) hippocampal subiculum, (b) CA1, and (c) CA2/3 at 28 days after ACA. Scale bar = 50 μm. APC366 significantly decreased neuronal degeneration in the hippocampal subiculum, CA1, and CA2/3 regions compared to the ACA + vehicle group at 28 days following ACA. Data are expressed as mean ± SD. *n* = 6/group. ANOVA, Tukey. **p < 0.001 compared to sham, *p < 0.05 compared to sham, &&*p* < 0.001 compared to ACA + vehicle

Based on better short-term outcomes, APC366 at dose of 50 μg was chosen for long-term studies. Long-term spatial learning and memory deficits following ACA were evaluated by Morris water maze test revealing prolonged escape latency and swim distance in ACA + vehicle animals compared to the sham group. APC366 markedly improved performance by decreasing escape latency and swim distance (Fig. 7a). In the probe trial, the percentage of probe quadrant duration was remarkably decreased in the ACA + vehicle group compared to the sham group. APC366 significantly increased the time that the ACA rats spent in the platform quadrant. There was no significance among groups in swim velocity (Fig. 7b). Consistently, APC366 significantly decreased the number of FJC-positive cells in the hippocampal subiculum, CA1, and CA2/3 regions compared to the ACA + vehicle group at 28 days following ACA (Fig. 8a, b).

### APC366 detection in the brain following intranasal administration

The MS/MS spectra of APC366 in the brain tissue showed a characteristic peak at m/z 441, which was similar to the APC366 standard ion pattern, suggesting the penetration of APC366 into the brain following its intranasal administration. Additional figure file demonstrates the detection of APC366 in the brain using (LC-MS/MS) detection system (Additional file 1).

### Inhibition of MC-tryptase exhibited anti-neuroinflammatory effect by suppressing the PAR-2/p-p38/NFκB pathway following ACA

**Fig. 9:**
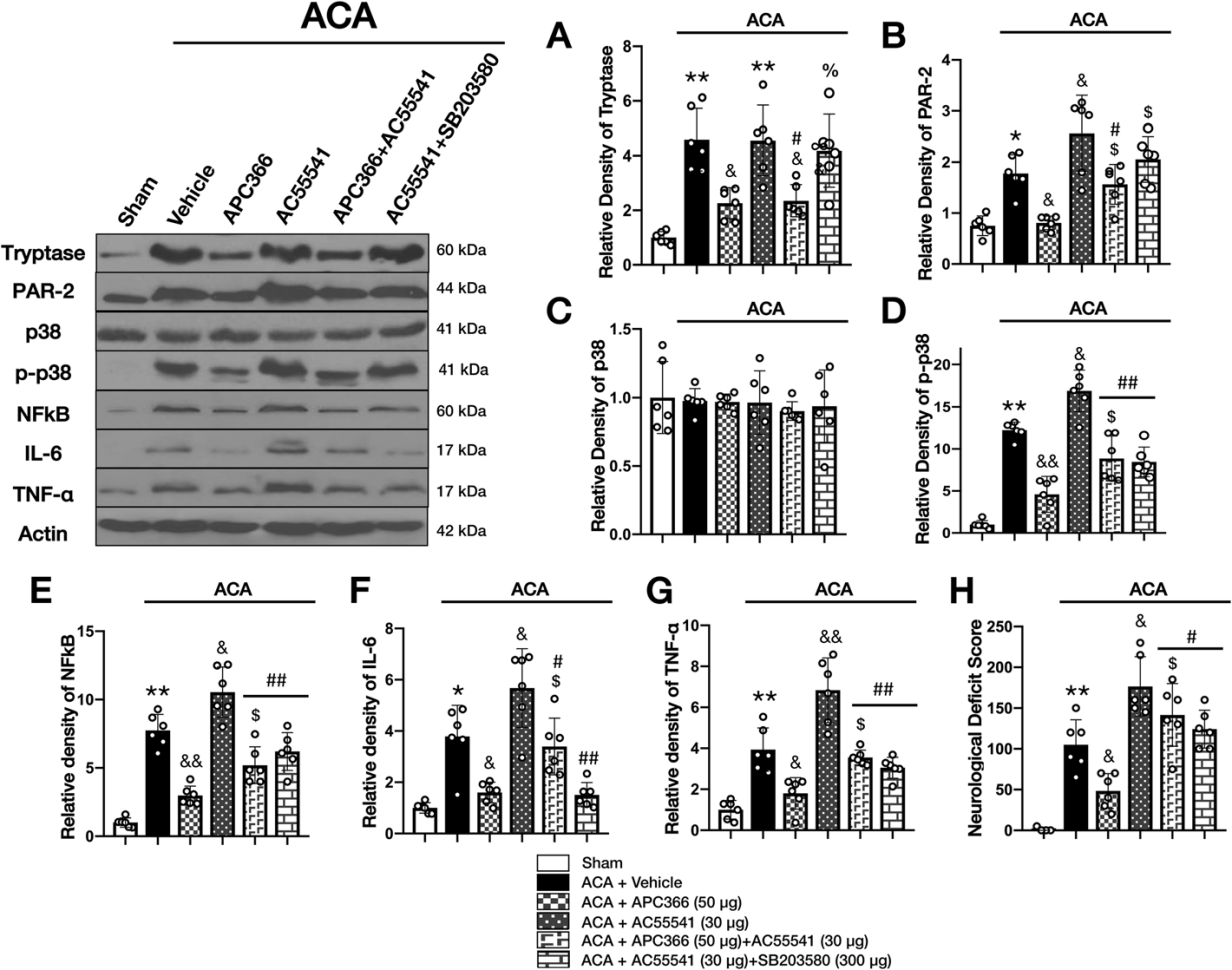
PAR-2 activation reversed the anti-neuroinflammatory effects of APC366 at 24 h after ACA. Representative western blot images and quantitative analysis of MC-tryptase (**a**), PAR-2 (**b**), p38 (**c**), p-p38 (**d**), NFκB (**e**), and proinflammatory cytokines (**f**, **g**) in the brain at 24 h following ACA. The levels of the pathway proteins were markedly increased following ACA. The inhibition of MC-tryptase significantly reduced PAR-2, p-p38 and NFκB, TNF-α, and IL-6 levels in rats treated with APC366 compared to the ACA + vehicle group. Further activation of PAR-2 by AC55541 caused higher expressions of p-p38, NFκB, and proinflammatory cytokines compared to the vehicle-treated ACA group. The co-administration of APC366 and AC55541 not only abolished the protective effect of APC366 in ACA rats, but also offset the detrimental effects of pharmacological activation of PAR-2 by AC55541, resulting in significantly lower levels of p-p38, NFkB, IL-6, and TNF-a compared to AC55541 alone. Selective p38 inhibitor, SB203580 reversed the aggravated neuroinflammation due to AC55541 by decreasing the expressions of p-p38, NFκB, IL-6, and TNF-α. **h** Neurologic deficit score at 24 h following ACA. The inhibition of MC-tryptase significantly improved neurologic function. This effect was reversed with the activation of PAR-2 by AC55541 at 24 h following ACA. The activation of PAR-2 by AC55541 alone significantly worsened neurological performance; however, this effect was rescued by the p-38 inhibitor, SB203580, compared to the ACA + vehicle group. Data are expressed as mean ± SD. *n* = 6/group. ANOVA, Tukey. **p < 0.001 compared to sham, *p < 0.05 compared to sham, &&*p* < 0.001 compared to ACA + vehicle, &*p* < 0.05 compared to ACA + vehicle, $*p* < 0.05 compared to ACA + APC366 (50 μg), %*p* < 0.05 compared to ACA + APC366 (50 μg) + AC55541 (30 μg), #*p* < 0.001 compared to ACA + AC55541 (30 μg), ##*p* < 0.05 compared to ACA + AC55541 (30 μg)

The levels of MC-tryptase, PAR-2, p-p38, NFκB, TNF-α, and IL-6 markedly increased at 24 h following ACA. Selective MC-tryptase inhibitor APC366 significantly reduced the protein levels of PAR-2, p-p38 and NFκB, TNF-α, and IL-6 levels compared to the ACA + vehicle group. The anti-neuroinflammatory effect of APC366 was abolished with selective PAR-2 activator AC55541, suggesting PAR-2-mediated MC-tryptase-induced neuroinflammation following ACA. Conversely, PAR-2 activation by AC55541 alone aggravated neuroinflammation, which was associated with higher expressions of p-p38, NFκB, and proinflammatory cytokines compared to the ACA + vehicle group. Interestingly, co-administration of APC366 and AC55541 not only abolished APC366 protective effects, but also offset the detrimental effects of pharmacological activation of PAR-2 by AC55541, resulting in significantly lower p-p38, NFkB, IL-6, and TNF-a compared to AC55541 alone. Moreover, the selective p38 inhibitor, SB203580, reversed the augmented neuroinflammation due to AC55541 alone by decreasing the expressions of p-p38, NFκB, IL-6, and TNF-α, confirming p-p38 as the downstream effector of PAR-2 activation by AC55541 following ACA (Fig. 9a–g, Additional file 2).

### Pharmacological activation of PAR-2 exacerbated neurologic function and abolished the beneficial effect of MC-tryptase inhibition following ACA

AC55541 alone markedly exacerbated neurological performance compared to the ACA + vehicle group, which was offset by the p-38 inhibitor, SB203580. The inhibition of MC-tryptase significantly improved the NDS which was reversed with the pharmacological activation of PAR-2 by AC55541 at 24 h following ACA. (Fig. 9h).

## Discussion

In this study, we focused on the role of brain MC-tryptase in neuroinflammation following CA-induced GCI. Our novel findings were as follows: (1) the expressions of MC-tryptase, PAR-2, p-p38, and NFκB were significantly increased in the brain over 72 h after ACA. (2) The intranasal administration of selective MC-tryptase inhibitor, APC366 [37, 38], 1 h after resuscitation significantly improved short- and long-term neurologic outcomes including cognitive functions after ACA. The neurobehavioral benefits were associated with less FJC-positive degenerating neurons in the hippocampus. The activation of PAR-2 with AC55541 further exacerbated the neurological impairments and hippocampal neuron damages after ACA. (3) APC366 significantly reduced brain expressions of MC-tryptase, PAR-2, p-p38, NFκB, IL-6, and TNF-α, which were reversed by AC55541. AC55541 alone, however, significantly aggravated the neuroinflammatory response by further increasing the protein levels of PAR-2, p-p38, NFκB, IL-6, and TNF-α. The proinflammatory effects were offset by the p-38 inhibitor, S203580. These results suggested that the neuroinflammatory effects of MC-tryptase were partly through activating the PAR-2/p38/NFκB signaling after ACA.

Brain MCs were appointed as the first responders to brain injury and initiators of the neuroinflammatory response [11, 39]. Emerging evidence has suggested the role of neuroinflammation in neurodegenerative diseases and cognitive and psychiatric disorders [12–14]. Particularly, the crosstalk between MCs and microglia, the two key immune cell populations which govern the neuroinflammatory response, has been implicated in neuroinflammation-induced conditions involving the central nervous system, including cognitive disorders [12, 40].

Increased MC-tryptase levels after brain injury, contributing to neuroinflammation-associated neurological deficits, were previously reported as well [33, 41, 42]. MC-tryptase was shown to potentiate the release of proinflammatory mediators from microglia, likely through the PAR-2 [16, 17].

PAR-2 belongs to a group of G protein-coupled seven transmembrane domain receptors that are activated by serine proteases which cleave the N-terminal receptor sequence at ^33^SKGR↓SLIGRL^42^ sites and expose the sequence of tethered ligand which is SLIGRL for rat PAR-2. The tethered ligand then binds to a site on the second extracellular loop of the receptor and triggers the receptor’s autoactivation which in turn results in the activation of signaling cascades such as the phosphorylation of mitogen-activated protein kinases [43]. Thrombin, the activator of all PARs except PAR-2, was previously shown to have an influence on ischemia-mediated neurodegeneration through PAR-1, PAR-3, and PAR-4 receptors after focal cerebral ischemia [44]. Among all, PAR-1 was particularly underlined in inflammation subsequent to ischemic brain injury. For example, inhibition of PAR-1 was shown to have anti-inflammatory and anti-apoptotic effects in a rabbit model of transient GCI induced by cardiopulmonary resuscitation [45]. However, authors did not evaluate whether microglia were involved in PAR-1 inhibition-mediated neuroprotection after CA-associated brain ischemia. On the other side, a proliferator effect of thrombin on microglia through PAR-1 receptor was suggested in another study [46]. Although PAR-4 activation was also mentioned once to potentiate the proinflammatory effects of thrombin on microglia, given the less understood function of PAR-4 relative to other PARs, evidence in the literature regarding the involvement of PAR-4 in any types of ischemic brain injury including cardiac arrest-associated brain ischemia is lacking.

PAR-2 is widely expressed in the central nervous system and the involvement of its activation in neurodegenerative diseases is well-established [47]. The activator effect of PAR-2 on microglia and astrocytes, resulting in a neuroinflammatory response has been demonstrated in earlier in vitro studies [17, 48]. In addition to its contribution to the activation of microglia and astrocytes, the activation of PAR-2 leading to neuronal death and blood brain barrier disruption has been proposed as well [49, 50]. However, evidence regarding PAR-2 expression in response to cerebral ischemia is limited and controversial as well. For example, PAR-2 expression was significantly upregulated in hippocampal slice cultures after being exposed to severe ischemia but downregulated following mild ischemia [51]. On the other hand, PAR-2 was markedly augmented in the ipsilateral hemisphere following focal cerebral ischemia [52]. In response to MC-tryptase, subsequent PAR-2 activation resulted in a pro-neuroinflammatory state [17, 47]. Due to the high hippocampal expression [53], the activation of PAR-2 negatively regulated learning processes and cognitive functions [54]. In the current work, for the first time, we showed increased expression of MC-tryptase and PAR-2 in the brain following CA-induced GCI. We also provided in vivo evidence demonstrating that PAR-2 expression in the brain was linked to MC-tryptase given the attenuated PAR-2 expression in response to the selective inhibition of MC-tryptase with APC366 following CA. Thus, our results were in line with previous data indicating the MC-tryptase-dependent expression of PAR-2 [37, 49]. In addition to the evidence suggesting linked expression of PAR-2 to MC-tryptase [37], the ability of MC-tryptase to upregulate the protein expression of PAR-2 [49] as well as a positive correlation between their expression levels were proposed in earlier studies [37, 55–57]. However, despite our extensive research, we could not encounter literature evidence unveiling how MC-tryptase acts as a regulator on PAR-2 protein expression [49]. We speculated that it may be an endogenous regulation of receptors to the changes in corresponding ligand levels. To be noted, APC366 is known to inactivate MC-tryptase in a time-dependent and irreversible manner and inhibited expression of MC-tryptase in subjects treated with APC366 was previously shown [37]. Consistent with the literature, we observed decreased protein expression of MC-tryptase in APC366-treated rats. As MC-tryptase also has the ability to stimulate MCs for degranulation, it is possible that APC366 may act in part by inhibiting the ability of MC-tryptase released from MCs to stimulate further MC degranulation, which results in decreased expression of MC-tryptase in APC366-treated animals compared to the vehicle group [58, 59].

In the present study, further increased expression of PAR-2 with AC55541 exaggerated neuroinflammation, exacerbated hippocampal neuronal degeneration, and neurologic deficits in the ACA rats. When administered concomitantly, AC55541 reversed the inhibitory effect of APC366 on PAR-2 expression and also abolished its reformative effect on post-CA neurologic outcomes as well as protein levels of PAR-2, p-38, and NFκB. These findings supported our hypothesis that the neuroprotective effect of APC366 was through inhibiting MC-tryptase/PAR-2/p-38/NFκB signaling. Interestingly, the co-administration of APC366 and AC55541 not only abolished the protective effects of APC366 in ACA rats, but also partially reversed the detrimental effects of pharmacological activation of PAR-2 by AC55541 after ACA. Given that lower protein level of PAR-2 receptors were associated with APC366, it may account for the partial counteract effect of APC366 against the deleterious effects of direct PAR-2 activation exogenously by AC55541.

Cerebral ischemia was previously shown to augment the protein expression levels of NFκB in a number of experimental transient ischemia models [60–63]. NFκB was also underlined as a downstream mediator leading to increased production of proinflammatory cytokines following GCI [63]. Similarly, p38 was involved in the inflammatory process following cerebral ischemia and inhibition of its activation was associated with smaller infarct volumes and improved neurologic functions [64]. Interestingly, p-p38 and NFκB were particularly responsible for the neuroinflammatory condition which occurred following the activation of microglial PAR-2 in response to MC-tryptase in vitro [17]. Consistently, we observed increased expressions of p-p38 and NFκB in the brain, which in turn resulted in enhanced levels of proinflammatory cytokines TNF-α and IL-6 following CA. Such elevation was suppressed by the inhibition of MC-tryptase but aggravated by the activation of PAR-2 with AC55541 alone. The exacerbated neuroinflammation by AC55541 was offset by the p38 inhibitor, SB203580, at 24 h following CA. Neurologic deficits were associated with the status of the neuroinflammation. The mortality rate of the present work was in line with previous reports evaluating ACA in rodents [65–67]

Several potential limitations should be considered while interpreting the results of the present study. For example, in this study, we focused on the role of MC-tryptase in neurologic dysfunction following CA, but not MC itself. The reason for this approach was the high number of biologically active mediators released upon activation and degranulation of cerebral MCs, which demonstrate their own particular effects in response to various stimuli. As expected, MC stabilizers inhibit MC degranulation and, thus, preclude the release of all MC-derived mediators. Indeed, improved cognitive functions were achieved by MC stabilizers in previous clinical [14] and experimental studies [12]; however, inhibition of which MC-derived mediator led to these better outcomes was not examined. As tryptase is the major secretory protein of MCs, with well-known inflammatory effects, we elected to allow MC degranulation, but then we selectively inhibited MC-tryptase instead of avoiding MC degranulation by using stabilizers to examine the potential mechanism behind MC-tryptase-associated neuroinflammation and neurologic dysfunction following CA. To this end, we have intranasally administered a selective MC-tryptase inhibitor (APC366) for treatment purposes after ACA. It should be noted that, in many fields, pharmacologic inhibitors usually have effects on other proteins, kinases, or systems as well. For example, APC366 has been reported to be a relatively selective MC-tryptase inhibitor given the observed inhibition of other trypsin-like serine proteases such as thrombin [68] as well as chymotrypsin-like serine proteases such as MC-chymase with the administration of APC366 in previous reports [69]. Therefore, the influence of APC366 on the inhibition of other proteases cannot be ignored while considering its reported in vivo effects on MC-tryptase. Nevertheless, we elected to proceed with APC366 to inhibit tryptase rather than more specific inhibitors such as specific tryptase silencing RNA (siRNA) or tryptase CRISPR (clustered regularly interspaced short palindromic repeats) knockout plasmids due to the following reasons: (1) intraventricular injection at 24–48 h prior to injury induction is a common route to deliver these specific siRNA or CRISPRs to the brain in rodent models of neurological diseases [22, 25, 29, 35, 70, 71], which could have further decreased the almost 70% ROCS success of our model given the potential complications due to the intraventricular injection procedure. (2) Intravenous injection of siRNA or CRISPR, as an alternative route would require much higher doses with high cost and would potentially involve systemic effects. Therefore, we chose to use an intranasal delivery of a specific tryptase inhibitor in our study, which may also be clinical translational. Importantly, one should note that, even though the results presented in the paper strongly suggest that the drugs indeed reached the brain through the nasal route of administration, there are no studies to date that confirm the ability of these drugs to cross the BBB. APC366 is a selective tryptase inhibitor of MC-tryptase that has been used in a variety of preclinical studies to investigate the pathological role of MC-tryptase so far [72–74]. Moreover, we have evaluated the potential beneficial effects of 2 different doses of APC366 in the setting of ACA. Eventually, we demonstrated successful penetration of APC366 to the brain via the intranasal route by spectrometric analysis and a tendency toward better treatment efficacy of 50 μg APC366 as compared with 150 μg APC366, which might have occurred due to the potential toxic effects of higher doses of APC366 in these highly vulnerable and resuscitated animals. Nevertheless, we were able to demonstrate that neurological benefits of inhibition of MC-tryptase were associated with less degenerating neurons within the hippocampus and lower brain tissue proinflammatory cytokine levels due to diminished activation of PAR-2. Lastly, as we mentioned earlier, further research is needed to investigate the other potential mechanisms underlying the neuroprotective effects of particular inhibition PAR-2 signaling after ACA, as its contribution to the inflammatory response via activated astrocytes as well as blood brain barrier disruption and neuronal death cannot be ignored.

Today, MC-tryptase inhibitors are widely used during daily medical practice for the management of MC-associated inflammatory conditions such as asthma, rhinitis, and conjunctivitis [75]. Consequently, emerging evidence from human clinical studies specifically points to inhibition of MCs in the treatment of neuroinflammation-associated cognitive dysfunction. We presented the first in vivo evidence regarding the deleterious effects of MC-tryptase-induced neuroinflammation on post-CA neurologic functions, which was at least partly mediated through the PAR-2/p-p38/NF-κB signaling pathway. Performing a single-sex study with male rats only is another possible limitation of the present work, which might have induced male bias and might potentially result with unintentionally deleterious results to the neglected sex and economic loss [76]. Based on our previous experience with male rats in the rodent model of ACA and the likelihood of increased sample size and increased costs due to the possibility of increased variability by using female rats have led us perform the study with male rats only. Currently, sex is accepted as a biological variable for preclinical studies [77, 78]. Hence, studying solely with male animals might have caused overlooking the results or side effects regarding female sex [79] and we underline that our results cannot be attributable to both sexes.

Based on the results of the current work and existing evidence from previous clinical and experimental research, we propose that inhibition of MC-tryptase and MC-tryptase-dependent activation of PAR-2 may present novel targeted therapeutics for the management of neurological dysfunction, particularly cognitive impairment after CA. Given the importance of CA in the field of emergency medicine and critical care, further preclinical and clinical studies are warranted to confirm our hypothesis.

## Conclusions

Inhibition of MC-tryptase-induced neuroinflammation remains a promising strategy to attenuate CA-associated neurocognitive dysfunction.

## Supplementary information

**Additional file 1.** Detection of APC366 in the brain with Liquid Chromatography–Mass Spectrometry detection system at 24 hours after its intranasal administration. jpeg. Legend: (A) The full scan mass spectrometry (MS) signal of APC366 detected from the APC366 standard showed characteristic peak at m/z 441; (B) MS/MS spectrum of precursor ion at m/z 441 from APC366 in the brain of APC366-treated rats; (C) APC366 standard with the similar fraction patterns.

**Additional file 2.** Full-length blots for time course and mechanism studies. docx. Figure 1 and 2 includes the full-length blots for western blot analysis regarding the time course and mechanism studies.

## Data Availability

The datasets generated and/or analyzed during the current study are available from the corresponding author on reasonable request.

## Abbreviations

ACA: Asphyxia-induced cardiac arrest
CA: Cardiac arrest
GCI: Global cerebral ischemia-reperfusion injury
IL-6: Interleukin 6
ROSC: Return of spontaneous circulation
MC: Mast cell
PAR-2: Protease-activated receptor-2
p-p38: Phosphorylated p38
TNF-α: Tumor necrosis factor-α
