# Supplementary figures and images for: Inhibition of mast cell tryptase attenuates neuroinflammation via PAR-2/p38/NFκB pathway following asphyxial cardiac arrest in rats
Source: J Neuroinflammation. 2020 May 4;17:144. doi: 10.1186/s12974-020-01808-2 (PMC7199326; doi:10.1186/s12974-020-01808-2)

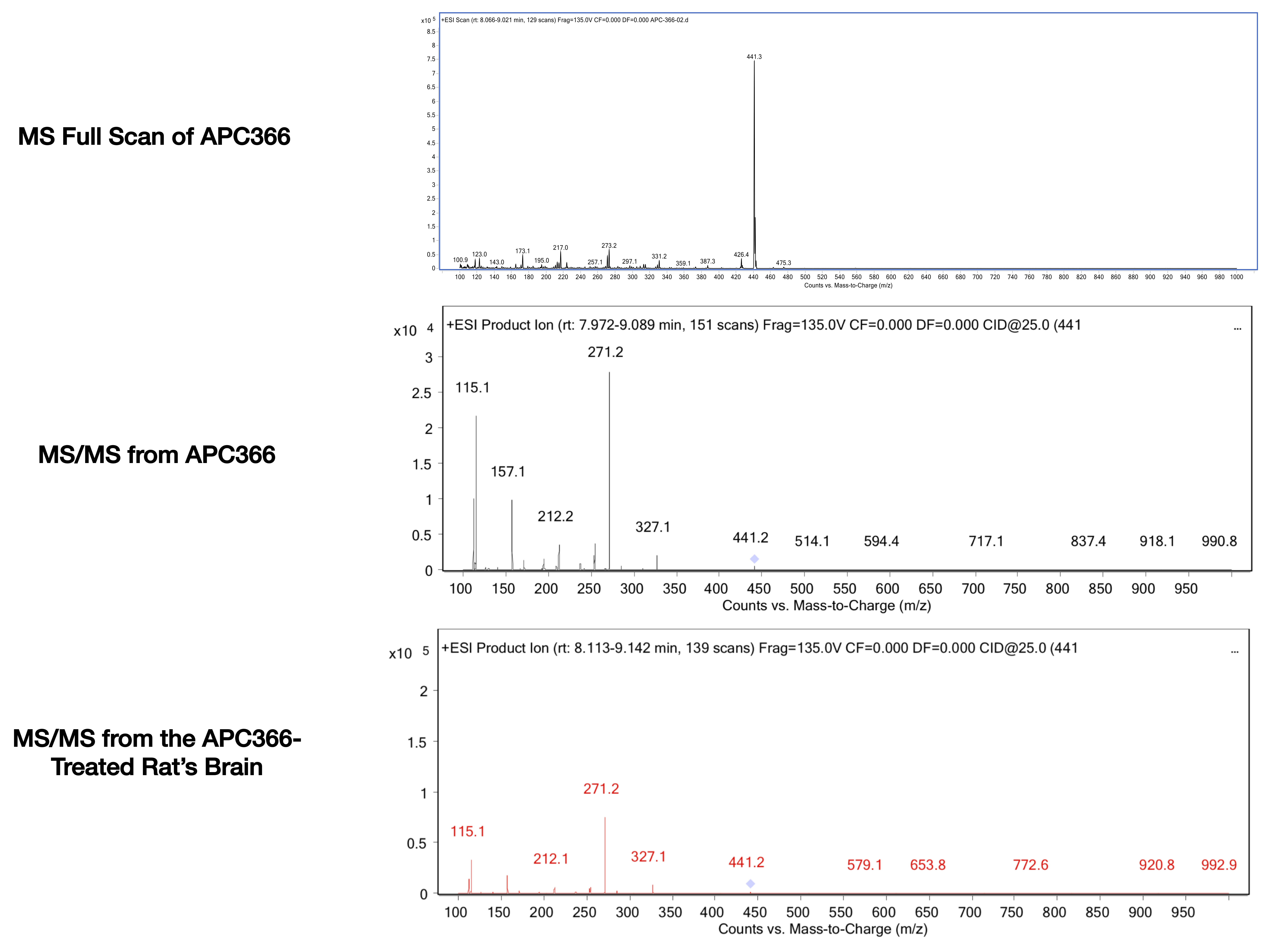

Supplement: Supplementary file 1 — Additional file 1. Detection of APC366 in the brain with Liquid Chromatography–Mass Spectrometry detection system at 24 hours after its intranasal administration. jpeg. Legend: (A) The full scan mass spectrometry (MS) signal of APC366 detected from the APC366 standard showed characteristic peak at m/z 441; (B) MS/MS spectrum of precursor ion at m/z 441 from APC366 in the brain of APC366-treated rats; (C) APC366 standard with the similar fraction patterns. [file 12974_2020_1808_MOESM1_ESM.jpg]
