# Supplementary material for: Inhibition of mast cell tryptase attenuates neuroinflammation via PAR-2/p38/NFκB pathway following asphyxial cardiac arrest in rats
Source: J Neuroinflammation. 2020 May 4;17:144. doi: 10.1186/s12974-020-01808-2 (PMC7199326; doi:10.1186/s12974-020-01808-2)
Supplement: Supplementary file 2 — Additional file 2. Full-length blots for time course and mechanism studies. docx. Figure 1 and 2 includes the full-length blots for western blot analysis regarding the time course and mechanism studies. [file 12974_2020_1808_MOESM2_ESM.docx]

**Sham 6 h 12 h 24 h 72 h**


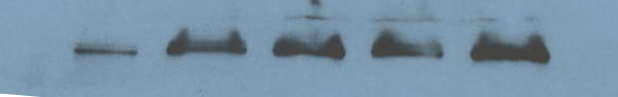


Tryptase60 kDA

60 kDA

PAR-2

44 kDA

**
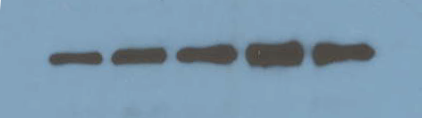
**


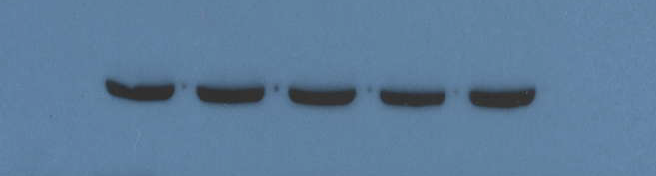


p38

41 kDA


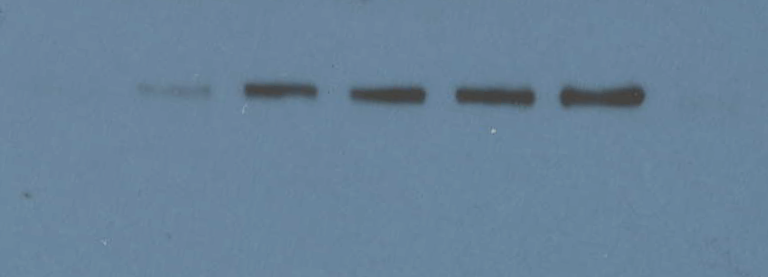


p-p38

41 kDA


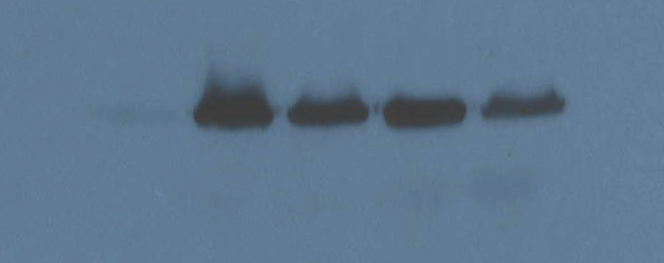


NFkB

60 kDA


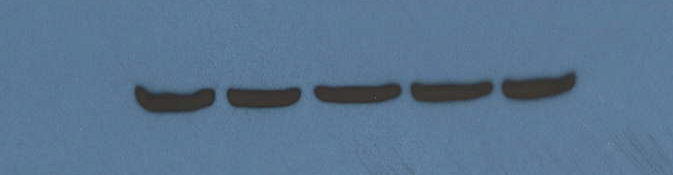


Actin

42 kDA

60 kDA

**Figure 1** Full length blots for time course experiment. Each lane was loaded with sample from a single animal from the group that is labeled in the blot.

**APC366+AC55541**

**APC366+AC55541**

**AC55541+SB203580**

**AC55541+SB203580**

**APC366**

**AC55541**

**APC366**

**Vehicle**

**AC55541**

**Sham**

**Vehicle**

**Sham**

Tryptase60 kDA

60 kDA

**
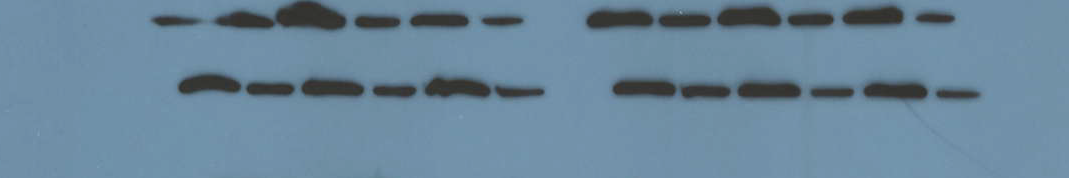
**

**
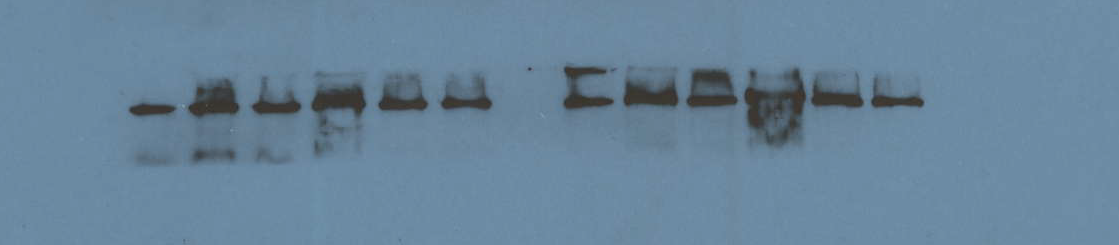
**

PAR-2

44 kDA

**
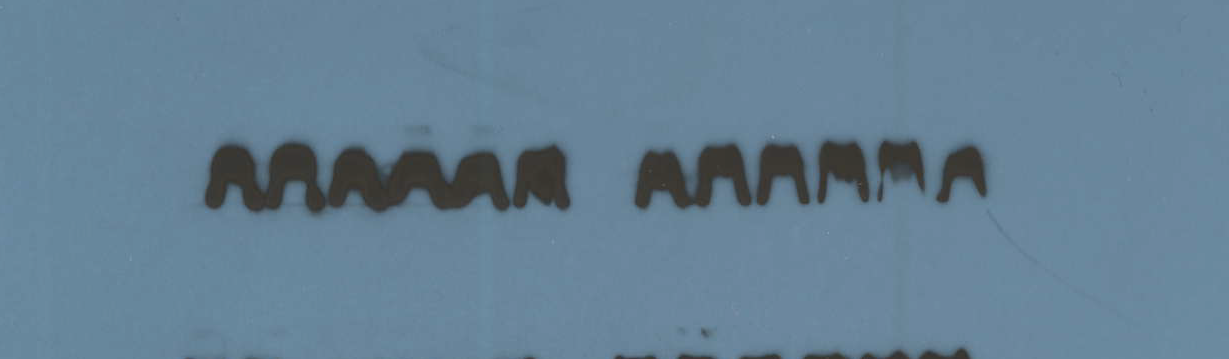
**

p-p38

41 kDA

p38

41 kDA

**
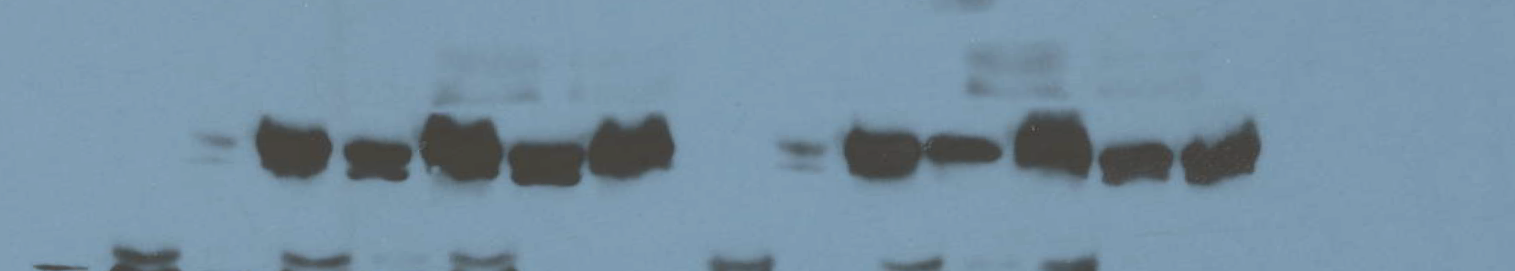
**

**
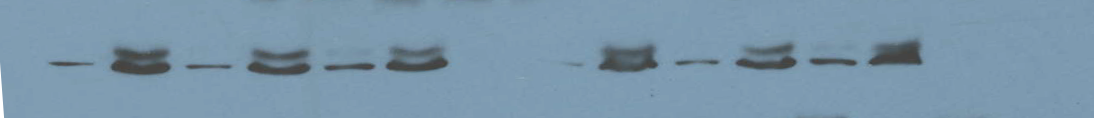
**

IL-6

17 kDA

NFkB

60 kDA

**
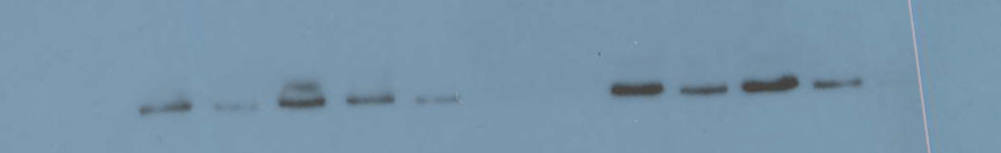
**

TNF-α

17 kDA

**
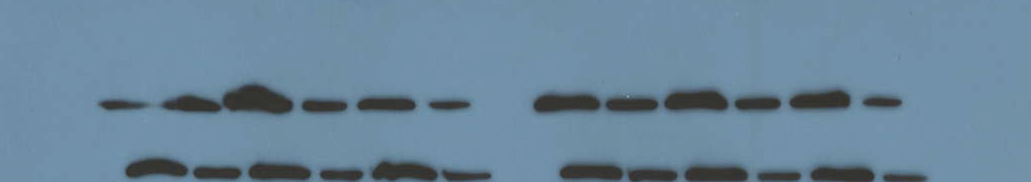
**

**
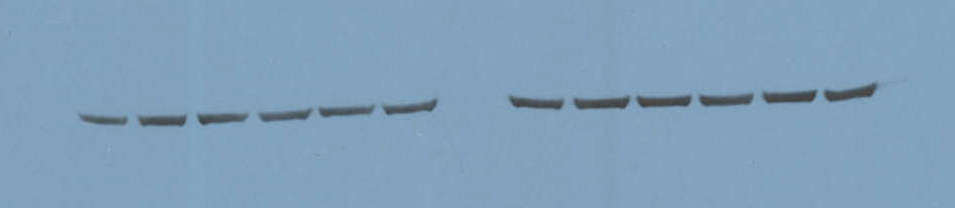
**

Actin

42 kDA

60 kDA

**Figure 2** Full length blots for mechanism experiment. Each lane was loaded with sample from a single animal from the group that is labeled in the blot.

**Figure 2** Full length blots for mechanism experiment. Each lane was loaded with sample from a single animal from the group that is labeled in the blot.
